# Recurrent Pleural Effusions Occurring in Association with Primary Pulmonary Amyloidosis

**DOI:** 10.1155/2015/421201

**Published:** 2015-09-02

**Authors:** Lauren Tada, Humayun Anjum, W. Kenneth Linville, Salim Surani

**Affiliations:** ^1^Corpus Christi Medical Center, 7002 William Drive, Corpus Christi, TX 78412, USA; ^2^Pulmonary & Critical Care, Bay Area Medical Center, 7002 William Drive, Corpus Christi, TX 78412, USA; ^3^Pathology Department, Bay Area Medical Center, 7002 William Drive, Corpus Christi, TX 78412, USA; ^4^Texas A&M University, 1177 West Wheeler Avenue, Aransas Pass, TX 78336, USA; ^5^University of North Texas, 1177 West Wheeler Avenue, Suite 1, Aransas Pass, TX 78336, USA

## Abstract

Recurrent pleural effusions occurring in association with immunoglobulin light chain amyloidosis and not associated with amyloid cardiomyopathy are rare. These portend an overall poor prognosis with mean survival time of approximately 1.8 months. We hereby report a case of a 59-year-old Caucasian female with recurrent pleural effusions and an ultimate diagnosis of pulmonary amyloidosis in association with plasma cell myeloma. The optimal treatment for recurrent pleural effusions in amyloidosis has not been determined; however, our patient responded to therapy with Cyclophosphamide-Bortezomib- (Velcade-) Dexamethasone (CyBorD) and had no repeat hospitalizations or recurrence of pleural effusion at four-month follow-up after initiation of therapy.

## 1. Introduction

Amyloidosis is a rare disorder of protein misfolding and deposition in various organs and tissues [1]. Primary amyloidosis often occurs in association with a plasma cell dyscrasia, whereas secondary amyloidosis tends to occur in association with longstanding chronic inflammatory diseases. The most common primary amyloidosis is immunoglobulin light chain (AL) amyloidosis. There are variable presentations which have been observed clinically with this disorder and commonly involved organ systems including renal, cardiac, gastrointestinal, neurologic, musculoskeletal, hematologic, and dermatologic [2]. Pulmonary manifestations are rare, and only 1-2% of patients with systemic amyloidosis develop persistent pleural effusions. This is important clinically because the presence of pleural effusions has been documented to portend an overall poor prognosis with mean survival time of 1.8 months when untreated [3]. Early diagnosis is critical because of these implications; however, a diagnostic challenge exists where a high clinical suspicion must be present and patients must undergo invasive biopsy and screening in order to secure an accurate diagnosis.

We hereby present a case report of a 59-year-old female with persistent pleural effusions and diagnosis of primary pulmonary amyloidosis in association with plasma cell myeloma.

## 2. Case Report

A 59-year-old Caucasian female with history of recurrent bilateral pleural effusions was admitted with worsening dyspnea and a nonproductive cough present over the course of one week. She had undergone outpatient right-sided thoracentesis on the day prior to admission, with drainage of 1500 mL of pleural fluid.

The recurrent pleural effusions had been occurring for three months prior to this presentation, and she had undergone thoracentesis twice for the right-sided pleural effusion and six times for the left-sided pleural effusion without any conclusive diagnosis. Results from all of thoracentesis procedures were suggestive of transudative effusions and cultures were negative.

**Figure 1:**
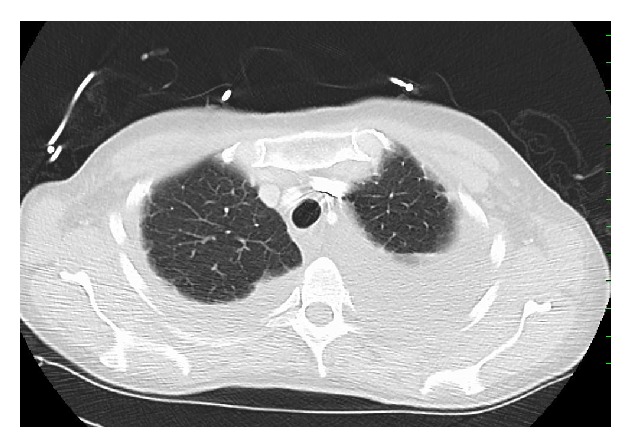
CT scan of the chest showing bilateral pleural effusions.

**Figure 2:**
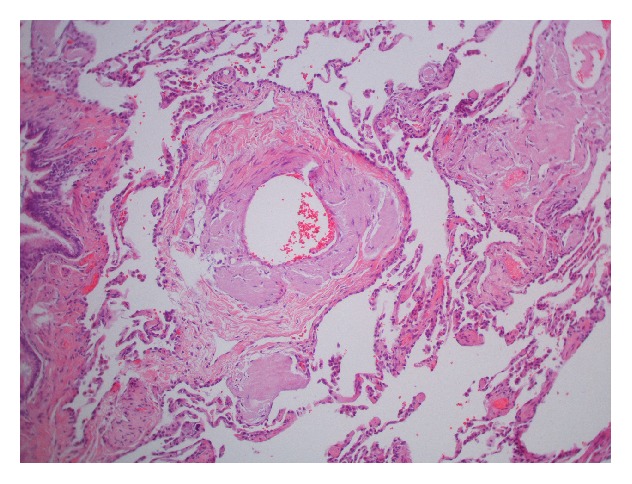
Thickened intrapulmonary vessel with adjacent interstitial eosinophilic amorphous material confirmed to be amyloid on Congo Red. (H&E, 100x).

**Figure 3 fig3:**
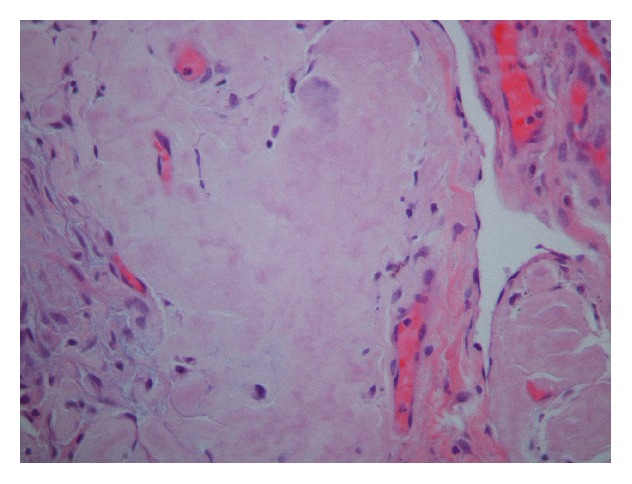
Intrapulmonary interstitial deposits of eosinophilic amorphous material that showed apple-green birefringence under polarized light on Congo Red stains (H&E, 400x).

**Figure 4:**
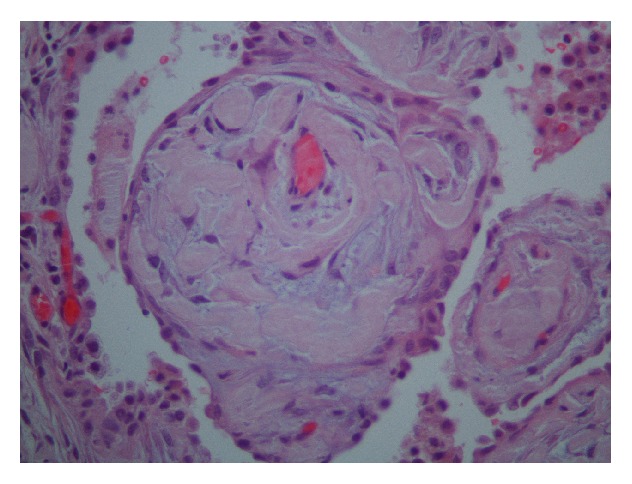
Pulmonary endothelial lined small vascular structure surrounded by amyloid deposits and marginated by a rim of reactive Type II pneumocytes (H&E, 400x).

On physical exam, this patient was mildly dyspneic but without retractions or accessory muscle use. There were decreased breath sounds at both lung bases. Temperature was 98.5°F, heart rate was 94/min, respiratory rate was 18/min, blood pressure was 97/55 mm Hg, and oxygen saturations were 99% on room air. Complete blood count and basic metabolic panel showed WBC 8.6 thou/*μ*L, Hb 14.1 g/dL, Hct 44.2 g/dL, Plt 356 thou/*μ*L, sodium of 140 mmol/L, potassium of 4 mmol/L, chloride of 101 mmol/L, bicarbonate 32 mmol/L, BUN 13 mg/dL, Creatinine 0.8 mg/dL, and glucose 112 mg/dL. Serum immunofixation electrophoresis showed small lambda monoclonal protein and no Bence-Jones proteinuria. Chest X-ray taken at the time of admission demonstrated a moderate to large left-sided pleural effusion as well as a right lower lobe consolidation. CT scan of chest showed bilateral pleural effusions, greater on the left side (Figure 1). During her hospitalization, the patient underwent thoracentesis which showed WBC 484 mm^3^, RBC 38 mm^3^, lymphocyte count of 99%, monocyte count of 1%, glucose of 106 mg/dL, total bilirubin of 2.4 gm/dL, lactate dehydrogenase (LDH) of 80 U/L, amylase 19 U/L, cholesterol of 45 mg/dL, triglyceride 17 mg/dL, and adenosine deaminase of 2.3 U/L, and the cultures for routine, acid fast bacilli, and fungus were negative. Cytology demonstrated benign findings with numerous lymphocytes present. The patient underwent biopsy of the left upper lobe of the lung as well as chemical pleurodesis for her recurrent left-sided pleural effusion. Lung biopsy showed diffuse pulmonary amyloidosis, and Congo Red staining was positive confirming the diagnosis (Figures 2 [Fig fig3]–4). Biopsy of the pleura was negative for any pathologic findings.

The patient underwent hematology/oncology evaluation. Echocardiogram showed left concentric ventricular hypertrophy. Rheumatoid factor and thyroid stimulating hormone levels were normal. Follow-up immunoglobulin and electron microscopy as well as bone marrow biopsy were performed and favored plasma cell myeloma over primary amyloidosis. There were 6% plasma cells on aspirate smears and 15–20% on CD138 immunohistochemical staining of biopsy and clot sections. Flow cytometry showed 1.4% of monoclonal plasma cells typical of a plasma cell dyscrasia.

The patient was started on Cyclophosphamide, Dexamethasone, and Bortezomib (Velcade) therapy and was discharged in stable condition with outpatient follow-up. She had no recurrent pleural effusions at four-month follow-up.

## 3. Discussion

Amyloidosis is a rare disorder that involves the formation of abnormal protein fibrils and deposition of these fibrils within various organs and tissues throughout the body. Amyloid fibrils are composed of low molecular weight protein subunits, which are normally soluble in the plasma [1, 4]. There are over 20 protein subunits recognized as those which may induce fibrillogenesis. All have a predominantly beta-pleated sheet configuration, which lends them the ability to bind Congo Red stain and demonstrate a characteristic apple-green birefringence by polarized microscopy.

Amyloidosis can be primary or secondary, systemic or localized, and inherited or acquired. AL amyloidosis is caused by immunoglobulin light chain fibril formation and deposition and is typically thought of as primary amyloidosis. It may occur alone or in association with a plasma cell dyscrasia. AA amyloidosis is caused by fibril formation by the acute phase reactant, amyloid A. This is typically seen in association with chronic diseases of inflammation and is therefore generally thought of as a secondary amyloidosis. Heritable forms of amyloidosis also occur and are generally referred to as AF amyloidosis, or familial amyloidosis. As the name implies, these disorders tend to be familial and associated with consistent patterns of clinical manifestation within families. The incidence of AL amyloidosis is unknown, but has been reported to be about 6 to 10 cases per million person-years [4]. The mean age of diagnosis is 64, and 65–70% of patients affected are male.

AL amyloidosis is a systemic disorder that presents with a variety of clinical manifestations depending on the predominantly affected organ system. Commonly involved organ systems include renal, cardiac, gastrointestinal, neurologic, musculoskeletal, hematologic, and dermatologic. Pulmonary involvement has rarely been reported, and published literature has suggested that only 1-2% of patients with systemic amyloidosis develop persistent pleural effusions. We have presented a patient with primary pulmonary AL amyloidosis whose initial presentation was for recurrent bilateral pleural effusions in order to contribute to the current literature for such patients. Our patient was also female and therefore provides information which may later serve as a reference for those who are in the minority of individuals affected by this disease.

Diagnostic criteria have been established by the Mayo Clinic and International Myeloma Working Group and require four criteria to be met for the diagnosis of AL amyloidosis. These include (1) amyloid deposition with distinct organ system involvement, (2) documentation of the presence of amyloid deposition by Congo Red staining, (3) evidence that the amyloid itself is formed by immunoglobulin light-chains, and (4) evidence of a monoclonal plasma cell proliferative disorder. This may be observed with serum or urine M protein, an abnormal serum free light chain ratio, or clonal plasma cells noted within the bone marrow [4]. Our patient met all four criteria and once again provided a unique presentation when compared with previously reported cases of AL amyloidosis.

In one retrospective analysis, medical records were examined for AL amyloidosis patients that had isolated respiratory system involvement between 1990 and 2011 [5]. Of the 13 cases identified during that time period, 9 male patients and 4 female patients were identified as having high mortality with a mean disease course of 46.5 months. Various presentations were recorded further indicating the diagnostic challenge that came with these cases. These included tracheal stenosis, bronchial stenosis, atelectasis, pulmonary nodules, lung consolidation, and lymph node enlargement.

Another published study compared persistent pleural effusions occurring in AL amyloidosis patients with cardiac involvement and those occurring in AL amyloidosis without cardiac involvement. Pleural involvement in this study indicated limited survival where the untreated persistent pleural effusions that occurred alone conferred a median survival of 1.8 months and where untreated persistent pleural effusions that occurred in association with cardiac involvement resulted in a mean survival of 6 months. These findings were found to be statistically significant with a* p* value of 0.031 [3]. Survival after chemotherapy and stem cell transplantation was noted to be comparable between groups with a mean survival of 21.8 months in the persistent pleural effusion group and 15.6 months in the group with persistent pleural effusion in association with cardiac involvement. These findings signal a poor prognosis where pleural effusions are present and untreated. It has been reported that parenchymal lung involvement occurs in approximately 28% of patients with AL amyloidosis; however, this does not appear to affect survival [3].

Various case reports have been published for pleural effusions presenting in cases of AL amyloidosis, and the presentations have been just as varied as amyloidosis has proven to be as a disease overall. There have been case reports of transudative as well as exudative pleural effusions (which appear to occur in about the same frequency) [6–8] and pleural effusions of various compositions. There have been different theories about the underlying pathogenesis of pleural effusions that occur in patients with AL amyloidosis based on these observations. The most common presentation of pleural effusions in AL amyloidosis patients occurs in association with amyloid-induced cardiomyopathy, and this suggests that there is pleural fluid accumulation that occurs as a result of ventricular dysfunction. Another possibility is that nephrotic syndrome causes low oncotic pressure in the serum and therefore formation of pleural effusions [3]. It has also been postulated that pleural effusions may result from impaired fluid resorption [9]. One case report of amyloidosis, which manifested with the parietal pleura being covered with brown nodules, seemed to support this theory, as the localized presence of amyloid would have resulted in occlusion of stomata on the parietal pleura where fluid resorption occurs. Still another theory is that severe inflammation that occurs from amyloid deposition might result in increased permeability of the pleural capillaries at the pleura. This has been given as one possible explanation for exudative pleural effusions that have been observed in AL amyloidosis [6].

It should be noted that pleural effusions might occur in AL amyloidosis as well as AA amyloidosis and Senile Systemic Amyloidosis [10–13]. Case reports have been published with pleural effusions present for each of these amyloidosis subtypes, and pleural fluid caused by AL amyloidosis has typically been documented to be composed of lymphocytic fluid with only two case reports of chylous fluid published in the current literature [14]. No clear association has been made for the pleural fluid composition and its occurrence, however. Our patient had pleural fluid of the lymphocytic type, and further studies will need to be undertaken to further elucidate this clinical finding.

Current treatment guidelines for AL amyloidosis involve the use of chemotherapy and autologous stem cell transplantation [15]. In the past, melphalan and prednisone were shown to prolong survival in patients with primary amyloidosis [16]. This combination treatment has been documented to increase median survival by approximately 17 months; however, there has not typically been regression of organ dysfunction or cure. The Amyloid Program at Boston University School of Medicine treated patients with systemic AL amyloidosis using high-dose intravenous melphalan and autologous stem cell transplantation and observed hematologic cure in 62% of patients with a 65% improvement in organ function providing some hope for improved survival in these patients [17]. The treatment of persistent pleural effusions from AL amyloidosis in particular has been more of a challenge, however, and generally requires optimizing cardiac filling pressure, symptomatic relief often with serial drainage as in our patient, and consideration of chemical pleurodesis for effusions that are refractory to other treatments. The optimal treatment for this specific condition has not been determined. There have been two case reports that have explored other chemotherapeutic regimens that have had good results for systemic amyloidosis patients with pleural effusions. In one study, a patient who had previously been receiving intermittent melphalan and prednisone therapy for 7 years was treated with vincristine, adriamycin, and dexamethasone (VAD) therapy with prevention of recurrence of his pleural effusions for the six months that he was on treatment, with return of his pleural effusions only after cessation of therapy [18]. Ultimately, this patient underwent chemical pleurodesis and continued melphalan and prednisone therapy with no increase in his pleural effusions at four-year follow-up.

In another published report, a patient that had diffuse parenchymal pulmonary amyloidosis was treated with melphalan, prednisolone, and Bortezomib chemotherapy [19]. This patient was subsequently noted to have normalization of her serum monoclonal protein levels as well as improvement over her overall pulmonary function and oxygenation with a 16.2% increase in vital capacity and 18.1% improvement in the diffusing capacity for carbon monoxide (DLCO). Similarly, there was a case report of a patient with recurrent pleural effusions and a subsequent diagnosis of AL amyloidosis which were treated with Cyclophosphamide-Bortezomib- (Velcade-) Dexamethasone (CyBorD) therapy with normalization of her serum protein immunoglobulin and no further recurrence of her pleural effusion at 8 months. Improvement in pulmonary function and oxygenation was also observed with this regimen. This is the treatment that our patient received after the diagnosis of AL amyloidosis, and, at the time of this report, there have been no repeat hospitalizations at 4 months [8].

## 4. Conclusion

Pleural effusions occur rarely in cases of systemic amyloidosis and have variable pleural fluid compositions. Because they portend a poor prognosis with a mean survival of 1.8 months when they occur in patients with AL amyloidosis, prompt diagnosis is critical in order to initiate treatment, which may improve pulmonary function and oxygenation and ultimately improve survival. This case provides an additional report of pleural effusions as they presented in a patient with AL amyloidosis and documents a current case being treated with CyBorD therapy.
